# Thematic Review of Endocrine Disruptors and Their Role in Shaping Pubertal Timing

**DOI:** 10.3390/children12010093

**Published:** 2025-01-16

**Authors:** And Demir, Adem Aydin, Atilla Büyükgebiz

**Affiliations:** 1 Pediatric Research Center, New Children’s Hospital, University of Helsinki, Helsinki University Hospital, 00290 Helsinki, Finland; 2 Department of Pediatrics, Faculty of Medicine, Dokuz Eylül University, 35340 İzmir, Türkiye; 3 Department of Pediatrics, Division of Pediatric Endocrinology, Demiroğlu Bilim University, 34394 Istanbul, Türkiye

**Keywords:** endocrine-disrupting compounds, pandemic, puberty, pollutants

## Abstract

This review examines the inconsistent effects of endocrine-disrupting chemicals (EDCs) and pollutants on pubertal timing, emphasizing the methodological challenges contributing to variability in findings. Data from nine key studies reveal that chemicals such as BPA, phthalates, and PFAS impact pubertal onset differently based on exposure timing, dosage, and sex. For instance, BPA is linked to earlier puberty in girls but delayed onset in boys, while other EDCs show mixed effects across populations. These discrepancies often arise from challenges in study design, such as the difficulty in establishing reliable control groups, accurately measuring exposures, and accounting for confounding factors like socioeconomic status, diet, and obesity. Sex-specific differences and environmental shifts during the COVID-19 pandemic, including increased indoor exposure and stress, further complicate the picture. These factors highlight the urgent need for more robust research methodologies, including standardized exposure assessments and longitudinal studies, to clarify the mechanisms driving these effects. Despite these challenges, the findings stress the importance of public health interventions, such as stricter EDC regulations, improved pollutant monitoring, and minimizing exposures during sensitive developmental windows. Addressing methodological gaps is crucial for producing reliable, actionable insights to protect adolescent development from the adverse effects of EDCs.

## 1. Introduction

Endocrine disruptors are defined as exogenous chemicals that can interfere with hormonal systems. They mimic, block, or alter the natural hormones in the body, leading to disruptions in the normal functions of the endocrine system. Endocrine-disrupting compounds (EDCs) can have both estrogenic and anti-estrogenic effects. These chemicals bind to hormone receptors, alter hormone production, disrupt hormone metabolism, and influence the transport and elimination of hormones from the body. Natural EDCs include phytoestrogens found in certain plants, which can mimic estrogen but do not accumulate in fat tissue. Synthetic EDCs are more prevalent and include pesticides, industrial chemicals like bisphenol A (BPA) and phthalates, and substances found in plastics, cosmetics, and other everyday products. These synthetic chemicals are particularly concerning because they can accumulate in fat tissue and persist in the environment, leading to long-term exposure. The wide range of health issues associated with EDC exposure include reproductive disorders (e.g., reduced fertility, early puberty), metabolic disorders (e.g., obesity, diabetes), neurological issues (e.g., autism, ADHD), and an increased risk of certain cancers. It also mentions the developmental effects of EDCs, particularly when exposure occurs during critical periods such as fetal development, infancy, and puberty.

Puberty is a critical phase in human growth and development, involving a complex cascade of physiological and hormonal changes that culminate in the transition from childhood to reproductive competence. The hypothalamic–pituitary–gonadal (HPG) axis initiates puberty. The HPG axis coordinates the production and secretion of primary sex hormones, mainly estrogen and testosterone, for the development of secondary sexual characteristics and reproductive organ maturation. The onset and progression of these events, collectively referred to as pubertal timing, differ substantially among individuals due to genetic predisposition in the context of environmental exposures and nutritional status. Interestingly, we have shown in a recent study that the timing of the onset of biochemical puberty is not different between the two sexes [1]. Regardless, this period has been characterized by a significant trend toward the earlier onset of clinical signs of puberty, especially in girls. Pubertal timing, primarily governed by genetics, is increasingly influenced by environmental factors, possibly including EDCs. This phenomenon has raised much concern and interest in developing methods to determine the timing onset of both biochemical and clinical puberty as well as to explain the root causes and health effects associated with this change in timing. For instance, in a semi-longitudinal study, we have shown that the quantification of nocturnal sleeptime urinary gonadotropin excretion can be used to predict the imminent appearance of clinical signs of puberty in girls [2].

EDCs represent a broad class of chemicals found in numerous consumer products, industrial processes, and environmental pollutants that can interfere with the endocrine system by either mimicking naturally produced hormones or blocking their effects, causing malfunctions at the molecular level [3]. Common examples of EDCs include BPA, phthalates, parabens, phenols, and per- and polyfluoroalkyl substances, or PFAS. These chemicals can be inhaled, ingested, and absorbed dermally, leading to ubiquitous human exposure.

There has been much interest in the relationship between endocrine-disrupting chemicals and the timing of puberty, which seems to be complex and multifaceted. For instance, BPA, which is widely used in food containers and packaging, has been associated with precocious puberty in females. Phthalates have antiandrogenic effects, with some reports suggesting that they could postpone pubertal development in males; phthalates are plasticizers with a wide range of uses. Parabens and phenols in personal care products also have endocrine-disrupting properties, which might influence the timing of puberty. PFAS, known to be very persistent in the environment, have been associated with delayed onset of puberty and other adverse health effects. In addition to EDCs, other environmental pollutants, like air pollutants, could implicate the disruption of pubertal timing. Precocious puberty that is more likely among urban populations is identified with long exposure to pollutants of air, in particular, PM and PAHs.

The COVID-19 pandemic has introduced a new set of variables to investigating pubertal timing. Lifestyle changes, high levels of stress, and increased time spent indoors might have potentially changed children’s exposure to EDCs and other environmental factors. There has been a significant increase in the number of reported cases of precocious puberty during this period, which requires further research on the effect of the pandemic on pubertal development. It is essential to understand fully the scope of environmental effects on the timing of pubertal development, as there may be life-long health implications. Early puberty is associated with a greater incidence of metabolic syndrome cardiovascular diseases, and psychological disorders. Delayed puberty can lead to growth and developmental concerns.

This review aims to synthesize recent findings from contemporary critical studies that explore the effects of EDCs and other environmental factors on the timing of the onset of puberty. Hence, this review focuses on the interactions between genetic predispositions and environmental exposures to bring out a comprehensive understanding of how these two factors interact in exerting influence on pubertal development and tries to project implications for public health and future research. These studies provide a truly varied perspective because the different populations, genders, and types of exposures studied can further add to our knowledge regarding the multifaceted quality of pubertal timing and its determinants.

## 2. Reviewed Literature

Study Selection: A review of the literature was conducted, focusing on the latest research regarding the relationship between environmental factors, mainly endocrine disruptors and pollutants, and the timing of puberty. Specifically, the inclusion criteria for the studies reviewed were as follows: 1. Studies that examine the impact of specific EDCs on pubertal timing, such as BPA, phthalates, and PFAS. 2. Studies that include both male and female subjects to explore possible gender-specific effects. 3. Research conducted with human subjects, especially children and adolescents. 4. Studies presenting detailed methodological approaches and rigorous statistical analyses.

The exclusion criteria were as follows: 1. Studies that did not directly address pubertal timing or focused on other unrelated health outcomes. 2. Animal studies that could not be directly extrapolated to human health. 3. Research with insufficient data or lacking methodological rigor. Based on the reviewed literature, we extracted the details of the study’s design, characteristics of the population, methods for assessment of exposure, measures of outcomes, and critical findings.

### 2.1. Gender-Specific Effects

***Greenspan et al. (2018)*** [4]

Population and Study Characteristics: This review article synthesizes findings from various epidemiologic studies conducted between 2016 and 2017. The populations covered in these studies include children and adolescents from different geographical regions (e.g., the U.S., China, Russia, and Europe), with both boys and girls being studied to examine the influence of EDCs on pubertal timing. The article includes data from longitudinal, cross-sectional, and case–control studies, primarily observational in nature.

Exposure Assessment Methods: Exposure to EDCs was assessed using biological samples such as blood, urine, and serum to measure levels of specific chemicals like BPA, phthalates, polybrominated diphenyl ethers (PBDEs), polychlorinated biphenyls (PCBs), and pesticides. These measures were often correlated with self-reported or clinically assessed pubertal milestones.

Outcome Measures: The primary outcomes included the timing of pubertal milestones such as thelarche, menarche, pubarche, and genital development in boys. These were evaluated through clinical assessments like Tanner staging, self-reported age of menarche, and testicular volume measurements.

Statistical Methods: Studies employed a range of statistical techniques, including multivariate regression models, to adjust for potential confounders and to assess the associations between EDC exposures and pubertal outcomes. These analyses often accounted for factors such as age, body mass index (BMI), and socioeconomic status.

Results: EDC effects on pubertal timing were found to be sex specific. For example, in girls, some studies found associations between higher BPA levels and delayed menarche, while in boys, moderate BPA levels were linked to earlier pubertal onset but slower progression to full maturity. Different EDCs had varying effects depending on the chemical structure and the timing of exposure. For instance, prenatal exposure to PBDEs was associated with delayed menarche in girls and earlier pubarche in boys. The effects of EDCs were influenced by exposure timing, with critical windows of susceptibility during prenatal and early childhood periods being particularly impactful.

***Berger et al. (2018)*** [5]

Population and Study Characteristics: The study was conducted within the CHAMACOS longitudinal cohort, involving 338 children (159 boys and 179 girls) born to mothers recruited from an agricultural community in Salinas Valley, California. The study focused on the association between prenatal exposure to high-molecular-weight phthalates and BPA with the timing of pubertal onset. The design is observational and longitudinal, with pubertal development assessed between ages 9 and 13 years.

Exposure Assessment Methods: Prenatal exposure to phthalates and BPA was quantified through urinary concentrations measured at two points during pregnancy. The study analyzed eight phthalate metabolites and BPA using high-performance liquid chromatography–tandem mass spectrometry (HPLC-MS/MS). These measurements were adjusted for urinary specific gravity to account for dilution differences.

Outcome Measures: The primary outcomes were the onset of pubertal milestones, including thelarche, pubarche, and menarche in girls, and gonadarche and pubarche in boys. Pubertal development was assessed every 9 months through clinical Tanner staging.

Statistical Methods: The study employed accelerated failure time (AFT) models to estimate the mean shifts in pubertal onset associated with each doubling of prenatal chemical exposure. Models were adjusted for maternal education, BMI, income, and other covariates. Interaction models were used to assess the modifying effects of childhood overweight/obesity status.

Results: Higher prenatal exposure to phthalates and BPA was associated with later pubertal milestones in girls. Specifically, di(2-ethylhexyl) phthalate (DEHP) and BPA were linked to delayed thelarche and menarche. The effect was more pronounced in normal-weight girls compared to those who were overweight or obese. Conversely, in boys, higher prenatal exposure to phthalates and BPA was associated with earlier pubertal onset, particularly in those who were overweight or obese. BPA was associated with earlier gonadarche regardless of weight status.

***Harley et al. (2019)*** [6]

Population and Study Characteristics: The study was based on the CHAMACOS longitudinal cohort, which followed 338 children (179 girls and 159 boys) in the Salinas Valley, California, from birth through adolescence. The study primarily involved Latino children from a farmworker community, with mothers generally having low educational attainment and income levels. Pubertal timing was assessed between ages 9 and 13 using clinical Tanner staging. The study is observational and longitudinal.

Exposure Assessment Methods: Exposure to phthalates, parabens, and phenols was assessed through urinary biomarkers measured during pregnancy and at age 9 in the children. Specific chemicals included monoethyl phthalate (MEP), methyl and propyl parabens, triclosan, benzophenone-3, and dichlorophenols. Biomarker concentrations were corrected for urinary creatinine levels.

Outcome Measures: The primary outcomes were the timing of pubertal milestones, specifically thelarche, pubarche, menarche in girls, and gonadarche in boys. These were assessed using clinical Tanner staging at regular intervals.

Statistical Methods: The study used accelerated failure time models to assess the relationship between chemical exposure and pubertal timing. Time ratios were converted to mean shifts in months to quantify changes in the timing of pubertal onset. Adjustments were made for maternal education, BMI, income, and other relevant covariates.

Results: In girls, prenatal exposure to MEP was associated with earlier pubarche, and prenatal triclosan and 2,4-dichlorophenol were linked to earlier menarche. Peripubertal exposure to methyl paraben was associated with earlier thelarche, pubarche, and menarche, while propyl paraben was linked to earlier pubarche. Conversely, 2,5-dichlorophenol was associated with later pubarche. In boys, there was limited evidence of association in boys, with only propyl paraben at peripubertal stages linked to earlier gonadarche.

***Fudvoye et al. (2019)*** [7]

Population and Study Characteristics: This review synthesizes recent data on the impact of EDCs on pubertal timing, focusing on both human epidemiological studies and animal models. It discusses secular trends in pubertal timing in girls and boys and the potential role of EDCs in these changes. The article draws on various studies conducted across different populations, emphasizing the increased incidence of central precocious puberty, particularly in girls.

Exposure Assessment Methods: The review examines exposure to EDCs through environmental pollutants, with specific attention to critical periods such as fetal and early postnatal life. EDC exposure is assessed through various methods, including epidemiological observations, biological sample analysis, and environmental monitoring.

Outcome Measures: The main outcomes evaluated include changes in pubertal timing, such as earlier or delayed onset of puberty, as well as reproductive health effects like hormonal disruptions. The review also explores neuroendocrine mechanisms affected by EDC exposure.

Statistical Methods: The article summarizes findings from various studies, employing diverse statistical methods to establish correlations between EDC exposure and changes in pubertal timing. It discusses trends in pubertal onset and the distribution of pubertal signs, though specific statistical techniques are not the focus.

Results: The review highlights the increasing incidence of central precocious puberty, especially in girls, and suggests a potential link between these trends and EDC exposure. It notes a skewed distribution in pubertal timing, with earlier onset of initial pubertal stages and delayed completion of puberty. The review also presents evidence from animal studies showing that exposure to EDCs during critical developmental periods can disrupt neuroendocrine control of puberty, leading to altered timing.

***Castiello et al. (2021)*** [8]

Population and Study Characteristics: This systematic review analyzed 13 epidemiological studies that investigated the relationship between exposure to non-persistent pesticides and puberty timing in children. The studies included both boys and girls, with sample sizes ranging from 30 to 12,727 participants. The studies were conducted in various countries, including Belgium, Denmark, Mexico, South Africa, China, and the United Kingdom. Most studies were cross-sectional, with some longitudinal and case–control designs, examining prenatal, postnatal, or childhood exposures to pesticides.

Exposure Assessment Methods: Exposure was assessed through various methods, including urinary concentrations of pesticide metabolites, questionnaires on pesticide use, and the area of residence as a proxy for exposure. The studies evaluated exposure to organophosphates, pyrethroids, herbicides, and fungicides, among others.

Outcome Measures: Puberty timing was assessed through age at menarche, Tanner staging for breast and genital development, and serum sex hormone levels (e.g., estradiol, testosterone, LH, FSH). Some studies focused on earlier or delayed sexual maturation as primary outcomes.

Statistical Methods: The reviewed studies employed a range of statistical methods to analyze the relationship between pesticide exposure and puberty timing, with adjustments for potential confounders. The quality of the evidence was assessed using the GRADE framework, and most studies were rated as providing low- to very low-quality evidence due to potential biases and small sample sizes.

Results: The review found that exposure to non-persistent pesticides was associated with altered puberty timing in several studies. In utero exposure to atrazine was linked to earlier menarche in girls, while exposure to organophosphates and pyrethroids was associated with delayed sexual maturation in both boys and girls. Conversely, pyrethroid exposure was also associated with pubertal advancement in boys. The evidence, however, was generally of low quality, with inconsistencies across studies.

#### Summary of Core Findings: Gender-Specific Effects

The gender-specific effects of EDCs on pubertal timing demonstrate consistent yet complex sex-specific patterns across various studies. Exposure to EDCs such as BPA, phthalates, pesticides, and parabens influences pubertal milestones differently in boys and girls. For girls, higher prenatal exposure to BPA and certain phthalates like di(2-ethylhexyl) phthalate (DEHP) generally delayed thelarche and menarche, while peripubertal exposure to parabens and some phenols was associated with earlier pubarche and menarche [5,6]. In boys, moderate BPA exposure advanced pubertal onset but often slowed progression [4]. Similarly, prenatal pesticide exposure showed diverse effects, with atrazine linked to earlier menarche in girls, while pyrethroids were associated with both delayed and advanced maturation in boys, depending on context [8]. Timing of exposure proved critical, with prenatal and early childhood periods being particularly sensitive windows [4,7].

The findings from these studies frequently align but also highlight methodological and contextual differences. For instance, studies from the CHAMACOS cohort [5,6] reported BPA and phthalates delaying girls’ pubertal milestones while advancing boys’, aligning with broader trends summarized in [4,7]. However, the review in [8] introduced inconsistencies, showing that pesticide exposure could both delay and advance boys’ pubertal timing. Supporting evidence from [7] highlighted neuroendocrine disruptions contributing to earlier pubertal onset in girls, consistent with findings on phenols and parabens in [6]. Contradictions, such as the opposing effects of pyrethroids [8], illustrate the complexity of EDC interactions and variability in study quality. Collectively, these studies underscore the interplay of chemical structure, timing, and individual susceptibility in driving gender-specific effects on pubertal timing.

### 2.2. Complex Interplay Between Genetic and Environmental Factors

***Binder et al. (2018)*** [9]

Population and Study Characteristics: This study was conducted within the Growth and Obesity Cohort Study (GOCS), involving 200 Latina girls from Santiago, Chile, born between 2002 and 2003. The study is longitudinal, assessing the association between urinary concentrations of phenols and phthalates at different pubertal stages (Tanner 1 and Tanner 4) and the timing of menarche. The girls were followed from childhood (ages 6.7 to 9.6 years at Tanner 1) into adolescence (ages 9.4 to 13.1 years at Tanner 4).

Exposure Assessment Methods: Exposure to phenols and phthalates was assessed by measuring 26 biomarkers in urine samples collected at Tanner 1 (before breast development) and Tanner 4 (during adolescence). The concentrations were quantified using high-performance liquid chromatography and mass spectrometry, and adjustments were made for specific gravity to account for urine dilution.

Outcome Measures: The primary outcome was the age at menarche, self-reported by the girls at each 6-month visit or during follow-up phone interviews. The study aimed to determine if exposure to these chemicals at different developmental stages influenced the timing of menarche.

Statistical Methods: The study used multivariable accelerated failure time (AFT) models to assess the association between biomarker concentrations and the timing of menarche. The models were adjusted for BMI Z-score and maternal education. The analysis also explored interactions between the Tanner stage and BMI with biomarker levels to account for potential effect modification.

Results: Higher concentrations of di(2-ethylhexyl) phthalate (DEHP) metabolites at Tanner 1 were associated with later menarche. Conversely, higher concentrations of 2,5-dichlorophenol and benzophenone-3 at Tanner 1 were linked to earlier menarche. Elevated concentrations of monomethyl phthalate (MMP) at Tanner 4 were associated with earlier menarche. The associations between other biomarkers and menarcheal timing were less consistent at Tanner 4. The study found that the associations between certain biomarkers (e.g., monoethyl phthalate and triclosan) and menarcheal timing were modified by BMI, with earlier menarche observed in overweight or obese girls with higher biomarker levels.

***Cirillo et al. (2021)*** [10]

Population and Study Characteristics: The study is based on the Child Health and Development Studies (CHDS) cohort, focusing on three generations: grandmothers (F0), their daughters (F1), and granddaughters (F2). The cohort includes 258 triads with complete data on DDT exposure and health outcomes. The study is a prospective, multi-generational cohort study that examines the impact of grandmaternal (F0) exposure to DDT during pregnancy on the granddaughter’s (F2) risk of early menarche and obesity in adulthood.

Exposure Assessment Methods: Exposure to DDT was assessed by measuring serum concentrations of o,p’-DDT, p,p’-DDT, and p,p’-DDE in the grandmothers (F0) during the perinatal period, primarily within a few days postpartum. The measurements were conducted using archived serum samples collected in the 1960s when DDT was still in use in the United States.

Outcome Measures: The primary outcomes were early menarche (≤11 years) and obesity (BMI ≥ 30 kg/m^2^) in the granddaughters (F2), assessed during a home visit in the 3Gs Study. Waist circumference was also measured as an additional indicator of obesity.

Statistical Methods: Log-linear models were used to estimate the associations between F0 serum DDT levels and F2 outcomes, adjusting for potential confounders, including F0 and F1 BMI, race, and F1 age at menarche. The analysis also considered interactions between F0 BMI and DDT levels.

Results: High levels of F0 o,p’-DDT were associated with a significantly increased risk of obesity in F2 granddaughters, particularly among those whose grandmothers had a normal BMI (OR, 2.6; 95% CI, 1.3, 6.7 for tertile 3 vs. tertile 1). In contrast, among grandmothers with higher BMI, this association was reversed, suggesting a potential modifying effect of grandmaternal obesity on DDT exposure outcomes. Higher F0 o,p’-DDT levels were also associated with a higher likelihood of early menarche in F2 granddaughters (OR, 2.1; 95% CI, 1.1, 3.9 for tertile 3 vs. tertile 1). This association was consistent regardless of F0 BMI.

***Faienza et al. (2022)*** [11]

Population and Study Characteristics: This review article explores the complex regulation of puberty timing, focusing on genetic, epigenetic, and environmental factors. It highlights the role of genes that control the gonadotropin-releasing hormone (GnRH) pulse generator, which initiates pubertal development. The article discusses conditions like central precocious puberty (CPP) and delayed puberty (DP), analyzing the roles of various genetic mutations, imprinted genes, and environmental influences.

Exposure Assessment Methods: The review examines the role of EDCs such as phthalates and BPA in altering puberty timing. It also evaluates genetic alterations, focusing on mutations in key genes such as MKRN3, DLK1, KISS1, and GNRHR. Epigenetic mechanisms, such as DNA methylation and histone modification, are also explored in relation to their effects on gene expression and pubertal timing.

Outcome Measures: The key outcomes are the onset of precocious puberty, delayed puberty, and their implications for long-term health. The review connects early or delayed puberty to broader health outcomes, including metabolic disorders and reproductive health, emphasizing how environmental and genetic factors contribute to these conditions.

Statistical Methods: Various studies analyzed in the review use genome-wide association studies (GWAS) to identify genetic variants linked to puberty timing. It also integrates evidence from epigenome-wide studies and transcriptomic analyses that evaluate the effects of environmental exposures and epigenetic modifications.

Results: The review highlights several critical genes that regulate puberty timing, including MKRN3 and DLK1, which play roles in inhibiting or promoting the onset of puberty. It also underscores the role of environmental factors, such as exposure to EDCs, in altering pubertal timing through epigenetic modifications. The authors suggest that epigenetic regulation may begin as early as fetal life, with long-term implications for puberty and reproductive health.

***Lu et al. (2022)*** [12]

Population and Study Characteristics: This study utilized genome-wide association study (GWAS) data from a cohort of 329,345 women to explore the genetic and environmental factors influencing the age at menarche (AAM). The study identified 9848 genes associated with AAM through a transcriptome-wide association study (TWAS), with a focus on the role of EDCs in altering the timing of puberty.

Exposure Assessment Methods: Genetic factors influencing AAM were examined using a TWAS, which integrated gene expression levels from the hypothalamus, pituitary gland, ovaries, uterus, and whole blood. The study also employed chemical–gene set enrichment analysis (CGSEA) to identify environmental chemicals (endocrine disruptors) associated with AAM. Data from the Comparative Toxicogenomics Database (CTD) provided the basis for evaluating the relationships between chemicals and gene expression.

Outcome Measures: The primary outcome was the timing of menarche, influenced by both genetic variants and environmental exposures. The study also highlighted the implications of early or delayed puberty for reproductive health, metabolic diseases, and cancer risk.

Statistical Methods: The study employed TWAS, CGSEA, and protein–protein interaction (PPI) network analysis to assess gene–environment interactions. Bayesian sparse linear-mixed models were used for the TWAS, while gene ontology (GO) and Kyoto Encyclopedia of Genes and Genomes (KEGG) enrichment analyses were applied to assess the biological significance of identified genes. The CGSEA identified significant relationships between 120 chemicals and AAM.

Results: The study identified 1580 significant genes associated with AAM across various tissues. Eleven genes were significantly associated with AAM across all five tissue types, including RBM6, PILRB, CPSF1, and HSD17B12. The CGSEA identified numerous chemicals significantly correlated with AAM, such as fluoxetine, ametryne, and isoflavones. The study also found that some of these chemicals could act as endocrine disruptors by interacting with genes involved in hormone metabolism and reproductive function.

***Freire et al. (2024)*** [13]

Population and Study Characteristics: The study analyzed data from 1223 children (579 girls and 644 boys) across 3 European birth cohorts: INMA (Spain), EDEN (France), and MoBa (Norway). The study examined the association between prenatal exposure to phthalates and synthetic phenols (including BPA, parabens, BP-3, and TCS) and pubertal development. Urinary biomarkers were measured from maternal urine samples collected during pregnancy, and pubertal development was assessed in the children at ages 7–12 years using the Pubertal Development Scale (PDS).

Exposure Assessment Methods: Maternal urinary metabolites of phthalates and synthetic phenols, including BPA, parabens, and triclosan (TCS), were measured in one or two urine samples collected during pregnancy. The study examined individual phthalates (DEP, DEHP, DiNP, etc.) and phenols for associations with pubertal outcomes.

Outcome Measures: The primary outcomes were overall pubertal onset, adrenarche, and gonadarche, as measured by the PDS. The study also evaluated the influence of prenatal chemical exposure on these outcomes, stratified by child BMI and developmental stages.

Statistical Methods: Mixed-effect Poisson regression, g-computation, and Bayesian Kernel Machine Regression (BKMR) were used to assess the associations between chemical exposures and pubertal development. Models adjusted for maternal education, pre-pregnancy BMI, and child BMI.

Results: Prenatal exposure to certain phthalates (DEHP, DiNP) was associated with a slightly higher probability of pubertal onset in boys, especially those with higher BMI. In contrast, exposure to BPA and parabens was linked to delayed puberty in boys. In girls, BPA and DEHP metabolites were associated with delayed gonadarche, particularly in those with normal or underweight BMI. The analysis suggested no overall effect of chemical mixtures on pubertal development.

#### Summary of Core Findings: Complex Interplay Between Genetic and Environmental Factors

These studies reveal the multifaceted relationships between genetic predispositions, epigenetic modifications, and environmental exposures in shaping puberty timing. Studies emphasize the dual influence of these factors, with genetic components such as MKRN3, DLK1, and other critical genes identified as regulators of pubertal onset, while environmental exposures, particularly to EDCs, modify these genetic effects through epigenetic mechanisms [9,10,11]. Binder et al. [9] highlighted stage-specific associations, where early exposure to phthalates and phenols either delayed or advanced menarche depending on the chemical and developmental timing. Cirillo et al. [10] extended this view across generations, linking maternal DDT exposure to early menarche and obesity in granddaughters, modulated by grandmaternal BMI. Freire et al. [13] confirmed these complex interactions by associating prenatal phthalates and BPA exposure with varying pubertal outcomes, influenced by child BMI and gender. Gender, while inherently a genetic factor, has been discussed in detail in a separate section above to allow for a focused exploration of its specific implications.

The findings across studies demonstrate significant overlap and complementarity, with genetic studies like Lu et al. [12] identifying specific gene–environment interactions and pathways influenced by EDCs, while Freire et al. [13] and Binder et al. [9] provided observational evidence of these effects in human cohorts. Supporting evidence from Faienza et al. [11] underscores how early-life exposures alter pubertal timing through epigenetic regulation, consistent with findings from Cirillo et al. [10] and Lu et al. [12] that link early menarche to broader health risks. However, inconsistencies exist, such as mixed results on whether certain phthalates delay or advance pubertal milestones [9,13]. Collectively, these studies highlight the intricate gene–environment interactions, underscoring that puberty timing results from the interplay between inherited genetic predispositions, environmental chemical exposures, and modifiable factors like BMI and developmental stage.

### 2.3. Effects of Persistent Environmental Pollutants

***Kehm et al. (2021)*** [14]

Population and Study Characteristics: The study analyzed data from the Columbia Center for Children’s Environmental Health (CCCEH) birth cohort, involving 196 Black and Hispanic girls born in New York City between 1998 and 2006. These girls were followed up from the prenatal period into adolescence, with assessments made on pubertal timing and body composition. The study is a prospective cohort study focusing on the impact of prenatal exposure to polycyclic aromatic hydrocarbons (PAHs) on pubertal development.

Exposure Assessment Methods: Prenatal exposure to PAHs was assessed using two methods: personal air monitoring data collected from backpacks worn by the mothers during the third trimester of pregnancy, and biomarkers of benzo[α]pyrene-DNA (BaP-DNA) adducts in umbilical cord blood. The air monitoring captured ambient levels of pyrene and eight higher-molecular-weight non-volatile carcinogenic PAHs, while the BaP-DNA adducts provided a measure of biologically effective dose.

Outcome Measures: The primary outcomes were the timing of pubertal milestones (growth spurt onset, breast development, and menarche) and body composition (BMI-for-age z-scores, waist-to-hip ratio, and percent body fat) assessed during adolescence (ages 11–20 years). Pubertal timing was self-reported by the girls, while body composition was measured during clinical visits.

Statistical Methods: The study used multivariable linear regression models to assess associations between prenatal PAH exposure and pubertal timing/body composition, adjusting for race/ethnicity, household public assistance at birth, birthweight, childhood BMI-for-age z-score, and age at the time of assessment. Inverse probability weights (IPW) were used to account for potential selection bias in analyses involving BaP-DNA adduct data.

Results: Higher prenatal exposure to PAHs was associated with delayed pubertal milestones. Girls in the highest tertile of ambient Σ8 PAH exposure had a 0.90-year delay in growth spurt onset and a 0.59-year delay in menarche compared to those in the lowest tertile. Similarly, higher BaP-DNA adduct levels were linked to delayed growth spurt and breast development onset. There were no significant associations between prenatal PAH exposure and adolescent BMI-for-age z-scores, waist-to-hip ratio, or percent body fat. The study found that prenatal PAH exposure did not appear to influence adolescent body composition, suggesting that the effects may be more relevant to pubertal timing than to long-term body composition.

***Oh et al. (2024)*** [15]

Population and Study Characteristics: The study utilized data from a retrospective cohort of 1,205,784 children aged six years, drawn from the Korea National Health Insurance Database. The cohort included 627,588 boys and 578,196 girls born between 2007 and 2009. The study followed these children until they reached 10 years of age for boys and 9 years of age for girls, assessing the incidence of precocious puberty (PP). The study is observational, non-interventional, and uses a nationwide cohort design.

Exposure Assessment Methods: Exposure to air pollution was assessed using long-term average concentrations of particulate matter (PM2.5 and PM10), sulfur dioxide (SO_2_), nitrogen dioxide (NO_2_), and ozone (O_3_) in the children’s residential areas. These data were derived from air quality monitoring stations and atmospheric simulation models, providing 12-, 24-, 36-, and 48-month moving averages of pollutant levels.

Outcome Measures: The primary outcome was the onset of precocious puberty, defined using ICD-10 codes and the administration of gonadotropin-releasing hormone agonist (GnRHa) treatments. The study measured the association between air pollution exposure and the risk of PP onset.

Statistical Methods: The analysis employed Cox proportional hazards models to estimate hazard ratios (HRs) for the onset of PP per unit increase in pollutant concentrations, adjusted for covariates such as income level, residential area, birth weight, and temperature. The study also conducted two-pollutant models to assess the combined effects of multiple pollutants.

Results: The study found significant associations between long-term exposure to PM2.5, PM10, SO_2_, and O_3_ and the onset of PP in girls. Specifically, for each unit increase in PM2.5, the HR was 1.019 (95% CI: 1.012, 1.027). Similar positive associations were observed for PM10, SO_2_, and O_3_. However, no significant associations were found for boys. The results remained consistent across different models, including two-pollutant analyses, indicating robust associations between air pollution exposure and PP in girls.

#### Summary of Core Findings: Effects of Persistent Environmental Pollutants

The studies in this section demonstrate distinct patterns based on pollutant type, exposure timing, and gender. Kehm et al. [14] showed that prenatal exposure to PAHs, as measured by ambient air monitoring and biomarkers in cord blood, significantly delayed pubertal milestones such as growth spurt onset and menarche in girls. This delay was consistent across various metrics of PAH exposure but did not appear to affect body composition in adolescence. Conversely, Oh et al. [15] found that long-term exposure to air pollutants such as PM2.5, PM10, SO_2_, and O_3_ was associated with an increased risk of precocious puberty (PP) in girls, with no significant associations observed in boys. These results suggest that the effects of persistent pollutants are both pollutant-specific and sex-specific, with differing impacts on pubertal timing and health outcomes depending on the exposure context.

The findings from these studies present complementary insights while also reflecting some contrasts. Kehm et al. [14] identified delayed puberty associated with prenatal exposure to PAHs, highlighting the importance of early developmental windows. In contrast, Oh et al. [15] documented earlier puberty linked to air pollution exposure during childhood, emphasizing the role of ongoing environmental exposures. Together, these studies underscore the varied mechanisms through which persistent pollutants influence puberty, with PAHs potentially disrupting endocrine pathways to delay pubertal onset, while air pollutants like PM2.5 and SO_2_ may accelerate puberty by triggering hormonal or inflammatory responses. The lack of consistent effects in boys across both studies suggests that girls may be more sensitive to these exposures, warranting further exploration of sex-specific vulnerabilities. Collectively, these findings highlight the complex interplay of pollutant types, exposure timing, and individual susceptibility in shaping pubertal development.

### 2.4. Impact on Pubertal Timing and Associated Health Implications

***Watkins et al. (2017)*** [16]

Population and Study Characteristics: This study investigated the impact of in utero exposure to phthalates and BPA on reproductive hormones and sexual maturation in peripubertal males. The study followed 109 male children aged 8–14 years from a Mexico City birth cohort, with maternal urinary samples collected during the first, second, and third trimesters of pregnancy to assess phthalate and BPA exposure.

Exposure Assessment Methods: Maternal urinary concentrations of phthalate metabolites and BPA were measured during each trimester using isotope dilution–liquid chromatography–tandem mass spectrometry (ID–LC–MS/MS). Specific gravity was used to adjust for urine dilution.

Outcome Measures: The primary outcomes included serum levels of testosterone, estradiol, dehydroepiandrosterone sulfate (DHEA-S), inhibin B, and sex hormone-binding globulin (SHBG), as well as sexual maturation indicators like Tanner staging and testicular volume in the male offspring.

Statistical Methods: Linear and logistic regression models were used to evaluate the associations between trimester-specific phthalate and BPA exposure and peripubertal hormone levels and sexual maturation outcomes. Sensitivity analyses were performed to assess the impact of exposure levels and rates of change across pregnancy.

Results: The study found that exposure to certain phthalates during the third trimester was associated with reduced odds of pubic hair development (Tanner stage > 1) and higher SHBG levels. First-trimester exposure to DEHP was associated with higher estradiol levels. No consistent associations were found with testosterone levels. The findings suggest that the timing of exposure during gestation is critical in determining its impact on male reproductive development.

***Lucaccioni et al. (2020)*** [17]

Population and Study Characteristics: This review examines the effects of EDCs on female puberty, focusing on how these chemicals influence the timing and progression of puberty. The review synthesizes findings from various human and animal studies, discussing how EDCs, particularly estrogen-mimicking endocrine disruptors (EEDs), affect pubertal development and potentially predispose individuals to breast cancer later in life.

Exposure Assessment Methods: The review discusses exposure to EDCs through various means, including ingestion, dermal absorption, and inhalation. The primary EDCs covered include BPA, DDT/DDE, dioxins, parabens, phthalates, and other chemicals commonly found in everyday products. Exposure assessments are based on the presence of these chemicals in biological samples and environmental monitoring.

Outcome Measures: The key outcomes reviewed include the timing of puberty, such as early onset of thelarche and menarche, and the development of secondary sexual characteristics. The review also explores the potential long-term effects of EDC exposure, such as an increased risk of breast cancer due to epigenetic changes and alterations in breast tissue development.

Statistical Methods: The article summarizes various studies, employing a range of statistical methods to establish correlations between EDC exposure and pubertal outcomes. The review itself does not focus on specific statistical techniques but rather on the overall evidence of associations.

Results: The review highlights that exposure to EDCs, particularly during critical windows like puberty, can lead to earlier onset of puberty and potentially predispose individuals to breast cancer. BPA and phthalates, for instance, are associated with premature thelarche and other early pubertal signs. The review also discusses how EEDs can alter breast tissue development through both direct and epigenetic mechanisms, increasing the risk of breast cancer later in life.

***Lughetti et al. (2020)*** [18]

Population and Study Characteristics: This review article examines the impact of EDCs on children’s health, particularly focusing on how these chemicals influence growth, pubertal timing, and the risk of developing various diseases later in life. The review synthesizes findings from a range of epidemiological studies and animal models, highlighting critical windows of susceptibility, including prenatal, postnatal, and pubertal periods.

Exposure Assessment Methods: The article discusses exposure to EDCs through ingestion, inhalation, and dermal absorption, emphasizing the ubiquitous presence of these chemicals in the environment. The EDCs reviewed include persistent organic pollutants (POPs), phthalates, BPA, and other industrial chemicals commonly found in consumer products.

Outcome Measures: The primary outcomes reviewed include alterations in pubertal timing, such as early onset of puberty, growth disruptions, thyroid dysfunction, and increased risk of metabolic syndrome. The review also explores the potential transgenerational effects of EDC exposure, highlighting the long-term health implications for future generations.

Statistical Methods: The review summarizes findings from various studies, employing a broad range of statistical methods to establish correlations between EDC exposure and health outcomes. The article does not focus on specific statistical techniques but emphasizes the challenges of linking exposure to long-term health effects due to the complexity of EDC actions and individual susceptibility.

Results: The review highlights that exposure to EDCs during critical developmental periods can lead to significant health issues, including altered pubertal timing, growth impairments, and an increased risk of obesity and metabolic disorders. EDCs such as BPA and phthalates are associated with early onset of puberty and thyroid dysfunction, which may predispose children to various endocrine and metabolic disorders.

***Czarnywojtek et al. (2021)*** [19]

Population and Study Characteristics: This review article synthesizes current knowledge on the impact of EDCs on the reproductive system. It includes studies involving both human and animal models, focusing on the effects of EDCs such as BPA, phthalates, polychlorinated biphenyls (PCBs), dioxins, pesticides, and phytoestrogens on male and female reproductive health. The article draws from various studies found in major medical databases until May 2021.

Exposure Assessment Methods: The exposure to EDCs was assessed through measurements in biological samples (e.g., blood, urine, tissue), environmental monitoring, and product usage analysis, highlighting how these chemicals are pervasive in daily life through diet, consumer products, and environmental contamination.

Outcome Measures: The primary outcomes evaluated include reproductive health effects such as premature puberty, infertility, hormonal disruptions, and reproductive cancers. The review also discusses mechanistic studies showing how EDCs interact with hormonal receptors and disrupt endocrine function.

Statistical Methods: The article does not focus on specific statistical methods but summarizes findings from various studies, some of which use epidemiological and mechanistic approaches to assess the impact of EDCs on reproductive health.

Results: The review highlights the widespread presence of EDCs in everyday products and their potential to cause significant reproductive health issues. BPA, for example, is associated with premature puberty and reproductive cancers, while phthalates and PCBs are linked to reduced sperm quality and other reproductive disorders. The evidence also indicates that EDCs can mimic or interfere with natural hormones, leading to disruptions in the hypothalamic–pituitary–gonadal axis, affecting both male and female reproductive health.

***Guth et al. (2021)*** [20]

Population and Study Characteristics: This cross-sectional study analyzed data from 382 Canadian girls aged 6–17 years, collected as part of the Canadian Health Measures Survey (2014–2015). The study population was diverse in terms of ethnicity and household income, with a majority of participants classified as having normal or underweight BMI. The study aimed to examine the association between urinary concentrations of parabens and serum reproductive hormone levels.

Exposure Assessment Methods: Paraben exposure was assessed through the measurement of urinary concentrations of four parabens: methylparaben, propylparaben, ethylparaben, and butylparaben. These concentrations were standardized for urinary creatinine to account for differences in urine dilution.

Outcome Measures: The primary outcomes were serum concentrations of reproductive hormones, including estradiol, progesterone, follicle-stimulating hormone (FSH), and luteinizing hormone (LH). These were measured using a clinical immunochemistry analyzer.

Statistical Methods: Multivariable linear regression models were employed to analyze the associations between urinary parabens and serum hormone levels, adjusting for potential confounders such as age, BMI, ethnicity, and household income. Sensitivity analyses were conducted using specific gravity to standardize urinary paraben concentrations and focusing on a younger subgroup of girls (ages 6–11 years).

Results: The study found that higher urinary concentrations of parabens were significantly associated with lower serum levels of estradiol, FSH, and LH, but not progesterone. A doubling in the sum of urinary parabens was associated with a 5.8% decrease in estradiol, a 4.2% decrease in FSH, and a 10.8% decrease in LH. These associations were consistent across individual parabens.

#### Summary of Core Findings: Health Implications of Altered Pubertal Timing

The selected studies highlight how EDCs affect pubertal development and long-term health. Watkins et al. [16] demonstrated that prenatal exposure to phthalates and BPA influenced hormone levels and sexual maturation in boys, with the timing of exposure being critical. Lucaccioni et al. [17] and Lughetti et al. [18] emphasized the effects of EDCs like BPA, phthalates, and POPs on early pubertal onset in girls and their potential to predispose individuals to metabolic and reproductive disorders, including breast cancer. Czarnywojtek et al. [19] expanded on these findings by detailing how EDCs disrupt the hypothalamic–pituitary–gonadal axis, causing hormonal imbalances, premature puberty, and infertility in both sexes. Guth et al. [20] provided evidence that paraben exposure was linked to reductions in estradiol, FSH, and LH levels in girls, suggesting a suppressive effect on reproductive hormones.

These studies collectively illustrate the pervasive and multifaceted influence of EDCs on pubertal and reproductive health, with substantial overlap and complementarity. Findings from Refs. [16,20] converge on the role of EDCs in altering hormone levels, although [16] focused on male sexual maturation while [20] examined female hormone suppression. Lucaccioni et al. [17] and Czarnywojtek et al. [19] aligned on the mechanistic insights into how EDCs mimic or interfere with natural hormones, leading to precocious puberty and long-term risks like cancer, supporting the broader conclusions of Lughetti et al. [18]. However, discrepancies exist, such as the differential impact of parabens and phthalates on male versus female development. Together, these findings underscore the importance of exposure timing, critical developmental windows, and individual susceptibility in determining the extent and nature of EDC-related health outcomes.

### 2.5. Future Research Directions

***Uldbjerg et al. (2022)*** [21]

Population and Study Characteristics: This systematic review and meta-analysis examined the effects of prenatal and postnatal exposure to EDCs on the timing of pubertal onset in girls and boys. The study included 52 publications for qualitative synthesis and 23 for meta-analysis, covering a wide range of EDCs, including phthalates, BPA, dioxins, flame retardants, parabens, and polychlorinated phenols. The studies involved diverse populations across various geographic regions and employed cohort, cross-sectional, and case-control designs.

Exposure Assessment Methods: EDC exposure was assessed through biomarkers in maternal or child biospecimens, with measurements taken during critical windows, including prenatal and postnatal periods. The study categorized EDCs into groups and assessed their impact on pubertal milestones such as thelarche, menarche, and pubarche in girls, and genital stage, testicular volume, and pubarche in boys.

Outcome Measures: The primary outcomes included the timing of pubertal milestones such as menarche, thelarche, pubarche, and genital stage. These were evaluated through clinical assessments, self-reports, and Tanner staging.

Statistical Methods: The meta-analysis involved pooling risk estimates from the included studies using random-effects models, with adjustments for confounding factors. The study also used qualitative trend synthesis to assess the direction of associations between EDC exposure and pubertal outcomes.

Results: The review found that postnatal exposure to phthalates was associated with earlier thelarche and later pubarche in girls, though the meta-analysis did not consistently confirm these findings across all studies. Prenatal exposure to phthalates and other EDCs showed weak and inconsistent associations with pubertal timing, with no clear evidence of critical windows of susceptibility. The overall evidence did not strongly support a significant impact of EDC exposure on pubertal timing, though some trends were noted.

***Taylor et al. (2023)*** [22]

Population and Study Characteristics: This systematic evidence map analyzed 98 human and 299 animal studies to examine the association between personal care products (PCPs) and pubertal timing. The studies included a wide range of populations, focusing on both boys and girls, with most human studies conducted in the United States, Denmark, China, Taiwan, and other countries. The studies varied in design, including cohort, cross-sectional, and case–control studies, and involved diverse racial/ethnic groups.

Exposure Assessment Methods: The exposure to PCPs and their chemical constituents, including phthalates, phenols, parabens, and PFAS, was assessed through various methods, including biomonitoring (e.g., urine, serum, breast milk) and questionnaires on PCP use. The studies also categorized exposures by chemical group and individual chemicals.

Outcome Measures: The primary outcomes reviewed included secondary sex characteristics (e.g., thelarche, pubarche), hormone levels (e.g., testosterone, estradiol), and clinical diagnoses of precocious puberty. These were measured using clinical assessments, self-reports, and laboratory analyses.

Statistical Methods: Various statistical methods were used across the studies, including logistic regression, linear regression, and mixture modeling, to evaluate the associations between PCP exposure and pubertal outcomes. Adjustments for potential confounders such as age, BMI, socioeconomic status, and race/ethnicity were commonly applied.

Results: The evidence map found that exposure to phthalates and phenols, particularly those in PCPs, was most frequently associated with changes in secondary sex characteristics and hormone levels. Phthalates like DEHP and phenols like BPA were consistently linked to earlier onset of puberty, particularly in girls. However, evidence varied, with some studies showing no significant associations or suggesting delayed puberty.

***Wang et al. (2024)*** [23]

Population and Study Characteristics: The review article examines various studies involving human populations and animal models to assess the impact of pharmaceutical and personal care products (PPCPs) on pubertal development. The human studies referenced include diverse populations across different countries, focusing on children and adolescents. The study types range from cross-sectional to longitudinal cohort studies, both observational and experimental.

Exposure Assessment Methods: Exposure to PPCPs, including antibiotics, psychotropic drugs, non-steroidal anti-inflammatory drugs (NSAIDs), synthetic estrogens, parabens, triclosan, and UV filters, was assessed through biological monitoring (e.g., urine, serum, and breast milk samples) and environmental monitoring (e.g., air, water, and dust). The concentrations of these chemicals were measured using advanced techniques such as high-performance liquid chromatography and mass spectrometry.

Outcome Measures: The primary outcomes measured were the timing of pubertal milestones, such as age at menarche, breast development, and pubic hair development, in human studies. In animal studies, outcomes included measures like vaginal opening (VO), preputial separation (PPS), and changes in hormone levels.

Statistical Methods: The studies reviewed employed various statistical methods, including regression analysis and meta-analysis, to determine associations between PPCP exposure and changes in pubertal timing. Specific statistical tools or software used were not detailed in the review.

Results: PPCPs, particularly synthetic estrogens, triclosan, and parabens, have been associated with altered pubertal timing in children, often leading to earlier onset in girls. However, results are inconsistent, with some studies showing no significant associations. Animal models consistently show that exposure to PPCPs can lead to both advanced and delayed puberty, depending on the type and timing of exposure. For example, synthetic estrogens like EE2 and parabens often accelerate puberty in female rodents, while some NSAIDs and psychotropic drugs delay pubertal onset in males.

#### Summary of Core Findings: Future Research Directions

The reviewed studies collectively emphasize the complexities and inconsistencies in understanding the impact of EDCs and PCPs on pubertal timing. Uldbjerg et al. [21] found mixed evidence on the effects of phthalates and other EDCs, with weak and inconsistent associations between prenatal exposure and pubertal milestones, although postnatal exposure showed some trends like earlier thelarche and later pubarche in girls. Taylor et al. [22] highlighted that some chemicals in PCPs, such as phthalates and phenols, are frequently linked to earlier puberty, especially in girls, though results varied across studies. Similarly, Wang et al. [23] reviewed the influence of PPCPs like synthetic estrogens, parabens, and triclosan, finding significant evidence of both advanced and delayed puberty depending on chemical type, timing, and population. While human studies showed variability, animal models often provided consistent evidence of altered pubertal timing.

These studies converge on identifying critical chemical groups like phthalates, phenols, and synthetic estrogens as significant contributors to changes in pubertal timing, but they also reveal key gaps. The variability in findings across Refs. [21,22,23] underscores the role of study design, population diversity, and chemical complexity in influencing results. Animal models in Ref. [23] consistently supported mechanistic insights on pubertal disruptions, complementing the mixed human evidence in Refs. [21,22]. The studies collectively suggest a need for standardized methodologies, longitudinal designs, and integrative approaches to clarify the nuanced relationships between chemical exposure, timing, and pubertal outcomes. Further research should explore critical windows of susceptibility, interactions between chemicals, and long-term health implications to address the gaps in current evidence.

## 3. Discussion

The relationship between pubertal development and EDCs is complex and shaped by interactions between genetic predispositions and environmental factors. While substantial evidence demonstrates the impact of EDCs on pubertal timing, significant inconsistencies across studies challenge definitive conclusions.

A primary methodological challenge is the difficulty in arranging and accessing reliable control groups, which reflects the inherent complexity of universal conditions affecting populations. Variations in socioeconomic status, diet, obesity, and environmental exposures make it nearly impossible to isolate unaffected groups, limiting the comparability and reproducibility of findings. Table 1 provides an overview of 21 studies investigating the effects of EDCs on pubertal timing, highlighting key findings and the variability in outcomes influenced by factors such as sex, BMI, and exposure timing.

The inconsistent effects of EDCs such as BPA, phthalates, and PFAS further complicate the picture. These chemicals have been associated with both delayed and accelerated puberty, depending on factors like chemical structure, timing of exposure, and sex. For instance, BPA exposure is linked to delayed menarche in girls but earlier pubertal milestones in boys. Similarly, phthalates and parabens show mixed effects that depend on dosage and developmental windows. These discrepancies highlight the urgent need for standardized protocols to address exposure assessment and methodological gaps.

Sex-specific differences add another layer of complexity. Estrogen-mimicking chemicals are more likely to cause earlier pubertal onset in girls, whereas boys experience delayed puberty, modified by factors like BMI. These findings demand nuanced exploration of the interplay between sex, environmental conditions, and lifestyle factors.

Genetic predispositions, such as mutations in MKRN3 and DLK1, also influence EDC effects, emphasizing the interdependence of biological and environmental factors. Transgenerational effects, as observed with DDT exposure, further demonstrate the long-lasting impacts of chemical exposures through epigenetic modifications.

The health implications of altered pubertal timing are significant, ranging from metabolic disorders and reproductive cancers to psychosocial challenges for early puberty, particularly in girls. Conversely, delayed puberty can lead to reproductive and endocrine dysfunction later in life. Addressing these risks requires public health strategies focused on reducing harmful exposures during sensitive developmental windows, such as the prenatal and early childhood periods.

Future research must prioritize overcoming the challenges of study design, particularly the arrangement of control groups, which reflects the natural complexity of universal conditions. Longitudinal studies and standardized methods are critical for identifying critical susceptibility periods and elucidating the mechanisms by which EDCs, genetics, and lifestyle factors interact. By addressing these inconsistencies and improving methodologies, researchers can provide actionable insights to mitigate the adverse effects of disrupted pubertal timing and protect adolescent development.

## 4. Conclusions

Inconsistencies in the effects of endocrine-disrupting chemicals (EDCs) on pubertal timing reflect both the inherent complexity of these interactions and significant methodological challenges. Chemicals such as BPA, phthalates, and PFAS exhibit highly variable impacts, influenced by chemical structure, dosage, timing of exposure, and sex. For example, BPA is linked to delayed menarche in girls but earlier puberty in boys, while other EDCs demonstrate mixed effects depending on context. These variations are further complicated by the universal complexity of conditions, which makes the arrangement of reliable control groups particularly difficult. Socioeconomic factors, obesity, and exposure to diverse environmental conditions act as confounders, limiting the generalizability of findings. Sex-specific differences further highlight the intricacy of EDC effects, with estrogen-mimicking chemicals often accelerating puberty in girls while delaying it in boys. Additionally, genetic predispositions, such as mutations in MKRN3 and DLK1, and transgenerational effects, like those seen with DDT exposure, add further complexity, revealing how environmental and biological factors interact to influence pubertal timing.

Resolving these inconsistencies requires addressing methodological limitations, such as standardized exposure assessments, and designing longitudinal studies capable of isolating critical susceptibility periods. Public health interventions must also focus on reducing harmful exposures during sensitive developmental windows. By overcoming these challenges through improved research and proactive measures, it is possible to better understand and mitigate the adverse effects of EDCs on adolescent development.

## Figures and Tables

**Table 1:** The table summarizes the 21 articles on endocrine-disrupting chemicals (EDCs) and pubertal timing. It includes study details, exposure types, outcome measures (e.g., pubertal milestones, hormone levels), and key findings, highlighting variability in effects by sex, BMI, and exposure timing.

Study	Population	Exposure	Outcome Measures	Key Findings
Greenspan et al. (2018) [4]	Various global cohorts (U.S., China, Europe, etc.)	BPA, phthalates, PCBs, pesticides	Tanner staging, pubertal milestones	BPA delayed menarche in girls; advanced pubarche in boys. Prenatal PBDEs delayed menarche in girls.
Berger et al. (2018) [5]	338 children (CHAMACOS cohort)	Prenatal phthalates, BPA	Pubertal milestones (thelarche, menarche, gonadarche)	Delayed pubertal milestones in girls; accelerated onset in boys with higher BMI.
Harley et al. (2019) [6]	338 Latino children (CHAMACOS cohort)	Phthalates, parabens, phenols	Pubertal milestones (thelarche, menarche, gonadarche)	Prenatal phthalates linked to earlier pubarche; parabens accelerated thelarche and menarche in girls.
Fudvoye et al. (2019) [7]	N/A (Review article)	EDCs (PCBs, pesticides, phthalates)	Pubertal timing, neuroendocrine mechanisms	EDCs disrupted neuroendocrine control; linked to central precocious puberty, especially in girls.
Castiello et al. (2021) [8]	13 studies (varied cohorts, n = 30–12,727)	Non-persistent pesticides	Age at menarche, Tanner staging, sex hormones	Atrazine linked to earlier menarche; organophosphates delayed maturation; mixed evidence quality.
Binder et al. (2018) [9]	200 Latina girls, Chile (GOCS cohort)	Phenols and phthalates in urine	Age at menarche	DEHP exposure delayed menarche; dichlorophenol exposure linked to earlier menarche; BMI modified effects.
Cirillo et al. (2021) [10]	258 triads, CHDS cohort (F0–F2 generations)	Grandmaternal DDT levels	Early menarche and obesity in F2 generation	F0 DDT exposure increased early menarche and obesity risk in F2, mediated by BMI and epigenetic changes.
Faienza et al. (2022) [11]	N/A (Review article)	Phthalates, BPA, genetic/epigenetic factors	Precocious and delayed puberty, metabolic effects	EDCs influenced puberty timing via epigenetic modifications. Key genes (MKRN3, DLK1) identified.
Lu et al. (2022) [12]	329,345 women (GWAS cohort)	Genetic factors, EDCs (fluoxetine, isoflavones)	Age at menarche (AAM)	Identified 1580 genes linked to AAM. Gene-chemical interactions disrupted hormonal pathways.
Freire et al. (2024) [13]	1223 children (INMA, EDEN, MoBa cohorts)	Maternal BPA, phthalates, parabens	Pubertal onset (adrenarche, gonadarche)	BPA delayed gonadarche in girls, mixed effects in boys. BMI and developmental stages influenced results.
Kehm et al. (2021) [14]	196 girls (CCCEH cohort, NYC)	Prenatal PAHs (ambient, biomarkers)	Pubertal milestones, body composition	Delayed menarche and growth spurts with high PAH exposure; no significant body composition effects.
Oh et al. (2024) [15]	1,205,784 Korean children	Air pollution (PM2.5, PM10, SO_2_, O_3_)	Precocious puberty	Air pollutants accelerated precocious puberty in girls but not boys.
Watkins et al. (2017) [16]	109 male children, Mexico City cohort	Prenatal phthalates, BPA	Tanner staging, hormone levels	Delayed pubic hair development with phthalates in T3; DEHP increased estradiol in T1.
Lucaccioni et al. (2020) [17]	N/A (Review article)	EDCs (BPA, phthalates, dioxins)	Pubertal timing, breast cancer risk	Earlier puberty and breast tissue changes linked to EDCs; potential long-term cancer risk.
Lughetti et al. (2020) [18]	N/A (Review article)	EDCs (BPA, POPs)	Growth, thyroid function, metabolic health	EDCs disrupted thyroid and metabolic health; linked to precocious puberty and obesity.
Czarnywojtek et al. (2021) [19]	N/A (Review article)	EDCs (BPA, PCBs, phytoestrogens)	Reproductive health, puberty timing	EDCs linked to premature puberty, reproductive cancers; disrupted HPG axis in both sexes.
Guth et al. (2021) [20]	382 Canadian girls	Urinary parabens	Serum reproductive hormones	Parabens reduced estradiol, FSH, and LH levels.
Uldbjerg et al. (2022) [21]	52 publications (meta-analysis)	Phthalates, BPA, flame retardants, parabens	Pubertal milestones (thelarche, menarche, pubarche)	Inconsistent evidence for critical windows; highlighted need for standardized methodologies.
Taylor et al. (2023) [22]	98 human and 299 animal studies	PCPs (phthalates, phenols, parabens)	Secondary sex characteristics, hormone levels	PCPs frequently linked to earlier puberty; variability in findings based on exposure type.
Wang et al. (2024) [23]	Various human and animal studies	PPCPs (synthetic estrogens, parabens)	Pubertal milestones (AAM, breast/pubic hair development)	Synthetic estrogens accelerated puberty; NSAIDs delayed male puberty; inconsistent human evidence.
